# Mental health dished up—the use of iPSC models in neuropsychiatric research

**DOI:** 10.1007/s00702-020-02197-9

**Published:** 2020-05-07

**Authors:** Rhiannon V. McNeill, Georg C. Ziegler, Franziska Radtke, Matthias Nieberler, Klaus-Peter Lesch, Sarah Kittel-Schneider

**Affiliations:** 1 Department of Psychiatry, Psychosomatic Medicine and Psychotherapy, University Hospital, University of Würzburg, Margarete-Höppel-Platz 1, 97080 Würzburg, Germany; 2 Department of Child and Adolescent Psychiatry, Psychosomatic Medicine and Psychotherapy University Hospital, University of Würzburg, Würzburg, Germany

**Keywords:** hiPSC, iPSC, Stem cells, Mental disorders, Affective disorders, ADHD

## Abstract

Genetic and molecular mechanisms that play a causal role in mental illnesses are challenging to elucidate, particularly as there is a lack of relevant in vitro and in vivo models. However, the advent of induced pluripotent stem cell (iPSC) technology has provided researchers with a novel toolbox. We conducted a systematic review using the PRISMA statement. A PubMed and Web of Science online search was performed (studies published between 2006–2020) using the following search strategy: hiPSC OR iPSC OR iPS OR stem cells AND schizophrenia disorder OR personality disorder OR antisocial personality disorder OR psychopathy OR bipolar disorder OR major depressive disorder OR obsessive compulsive disorder OR anxiety disorder OR substance use disorder OR alcohol use disorder OR nicotine use disorder OR opioid use disorder OR eating disorder OR anorexia nervosa OR attention-deficit/hyperactivity disorder OR gaming disorder. Using the above search criteria, a total of 3515 studies were found. After screening, a final total of 56 studies were deemed eligible for inclusion in our study. Using iPSC technology, psychiatric disease can be studied in the context of a patient’s own unique genetic background. This has allowed great strides to be made into uncovering the etiology of psychiatric disease, as well as providing a unique paradigm for drug testing. However, there is a lack of data for certain psychiatric disorders and several limitations to present iPSC-based studies, leading us to discuss how this field may progress in the next years to increase its utility in the battle to understand psychiatric disease.

## Introduction

Psychiatric disorders are currently one of the most challenging diseases to treat. Somatic disorders such as cancer have shown significant progress in the hunt for effective therapeutics, whereas the failure rate for novel psychopharmacological agents in drug development and clinical trials remains high (Arnerić et al. 2018; Cummings 2018; Kinch 2015). This is partly due to a lack of suitable in vitro and in vivo models for drug testing, as central nervous system tissue is difficult to access (Cummings 2018). Even when precious tissue can be accessed (e.g. post-mortem brain tissue), its use is limited by factors such as terminal cellular differentiation (no propagation possible), end-stage pathology, lack of adjustable experimental conditions, and the confounding effects of long-term medication and patient lifestyle (Silva and Haggarty 2019). Research has therefore relied heavily on animal models, only introducing the human context later at the clinical trial phase, which presents a number of inherent problems. Psychiatric disorders are by nature highly complex with multiple subjective symptoms, making the extrapolation of human phenotypes highly difficult (Nestler and Hyman 2010), and large neuroanatomical differences exist between humans and animals (Seok et al. 2013). This was highlighted in a recent review by Logan et al. (2019), who observed that in multiple studies of genetically engineered mice expressing human microcephaly-related gene mutations, the reduced brain size observed in humans was not able to be recapitulated (Logan et al. 2019).

In 2006, Takahashi and Yamanaka revolutionized the study of psychiatric disease by introducing pluripotent stem cell technology, which helped solve many of the problems associated with the use of animal models (Takahashi and Yamanaka 2006). Using a combination of only four transcription factors they demonstrated that human fibroblast cells could be transduced to form induced pluripotent stem cells (iPSCs), which were capable of forming all 3 germ layers and therefore had the potential to differentiate into any cell type of the body (Takahashi and Yamanaka 2006). Since then, it has been demonstrated that iPSCs can form neural progenitor cells (NPCs) using defined growth factors, resulting in homogenous, expandable and self-renewable cultures (Chambers et al. 2009; Cheng et al. 2017; Koch et al. 2009). Moreover, it was found that these cultures could be further stimulated to differentiate into various CNS cell types, including astrocytes and functionally active neurons (Muratore et al. 2014; Silva et al. 2016).

Several previous reviews have been published regarding iPSC-derived models of neuropsychiatric disorders, with early reviews including only a few published original studies and giving more an outlook on the promises and pitfalls of this new technology (for examples see (Brennand and Gage 2012; Dolmetsch and Geschwind 2011; Tobe et al. 2011; Vaccarino et al. 2011). As more research was published using iPSC-derived neuronal cells from patients with neuropsychiatric disorders, more specific reviews emerged. Initial research and reviews focused on neurodegenerative disorders such as Alzheimer’s disease (Essayan-Perez et al. 2019; Goldstein et al. 2015; Ooi et al. 2013), Parkinson’s disease (Parmar et al. 2020; Schwamborn 2018) and Huntington’s disease (Benraiss and Goldman 2011; Golas and Sander 2016; Wu et al. 2019). The rationale was that stem cell-based therapies might be easier to implement in these more defined diseases than in complex mental illnesses such as schizophrenia or major depression. However, there were several early studies and reviews on autism spectrum disorder (Aigner et al. 2014; Freitas et al. 2014; Russo et al. 2019) and schizophrenia (Brennand and Gage 2011; Brennand et al. 2014; Hoffman et al. 2019; Watmuff et al. 2016), as well as rare research on syndromic, monogenetic and/or chromosomal aberration disorders that can cause psychiatric phenotypes such as 22q11.2 microdeletion syndrome which is strongly associated with autism spectrum disorder, schizophrenia but also ADHD and affective disorders (Drew et al. 2011), Down syndrome (Faundez et al. 2018), Fragile X Syndrome (Faundez et al. 2018) and Timothy’s syndrome (Coskun and Lombardo 2016).

As iPSC technology developed and became more accessible, researchers began to focus on less well-defined psychiatric diseases, such as bipolar disorder (Hoffmann et al. 2018; Miller and Kelsoe 2017; O'Shea and McInnis 2015; Viswanath et al. 2015; Watmuff et al. 2016), alcohol use disorder (Prytkova et al. 2018) and anorexia nervosa (Maussion et al. 2019). There has also been a great interest in the use of 3D brain organoids instead of 2D neuronal cells, and the use of CRISPR/cas9 to genetically edit iPSC-derived models, which have been extensively reviewed elsewhere (Korhonen et al. 2018; Lee et al. 2017; Rehbach et al. 2020; Tian et al. 2020). In this systematic review, we aim to present an update of iPSC-based research into already relatively well-studied polygenic psychiatric disorders, less focusing on the syndromic and monogenetic forms, and review recent novel data on less well-studied disorders. Lastly, we will describe the present limitations of iPSC-based studies, and discuss how researchers can work to overcome them.

## Methods

Our systematic review was performed according to the *P*referred *R*eporting *I*tems for *S*ystematic reviews and *M*eta-*A*nalyses (*PRISMA*) statement (Moher et al. 2009). We conducted an online PubMed (on the 25/3/2020) and Web of Science (19/4/2020) search using the following search strategy: hiPSC [ti] OR iPSC [ti] OR iPS [ti] OR stem cells [ti] AND schizophrenia disorder OR personality disorder OR antisocial personality disorder OR psychopathy OR bipolar disorder OR major depressive disorder OR obsessive compulsive disorder OR anxiety disorder OR substance use disorder OR alcohol use disorder OR nicotine use disorder OR opioid use disorder OR eating disorder OR anorexia nervosa OR attention-deficit/hyperactivity disorder OR gaming disorder. Additional search criterion was studies must have been published between 2006 and 2020. Papers were initially screened by title only, and excluded if deemed not relevant because the study was not on iPSCs, not written in the English language, or no abstract was available. In a second round of screening, full-text papers were assessed for eligibility, and excluded if not relevant to the topic. We excluded reviews and meta-analysis and meeting abstracts as well as stem cell studies derived from animal models, studies on rare monogenetic disorders/syndromes and articles reporting protocols of hiPSC differentiation without including the generation of a disorder- and patient-specific iPSC cell line as well as study protocols of planned studies without actual results. This was firstly done subdivided into the disorder entities by the co-authors (GZ, FR, MN, KPL, SKS) and then again independently for all topics from one co-author (RVMcN) as well as the Web of Science search of another co-author (SKS).

## Results

**Fig. 1:**
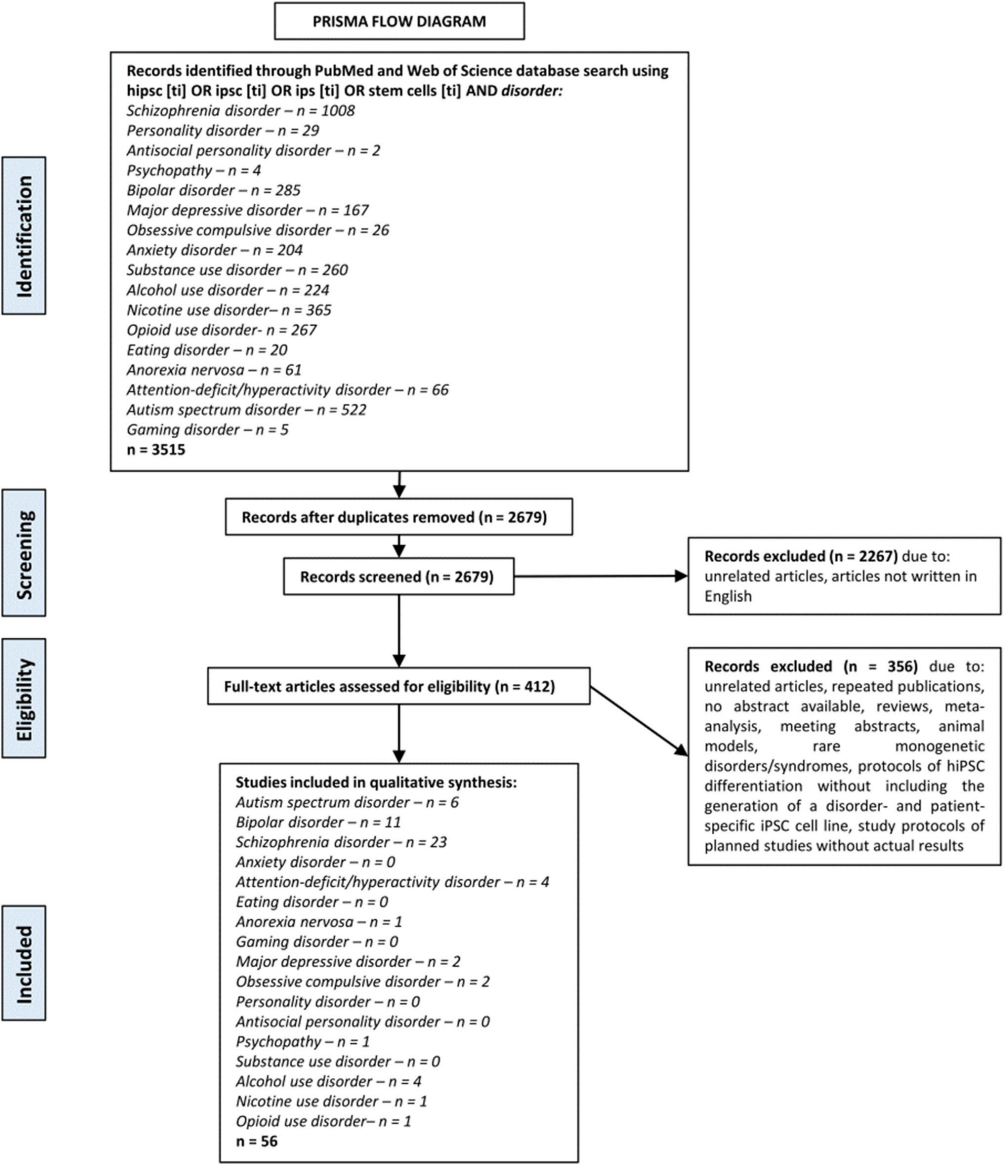
PRISMA flow diagram

A total of 3515 records were retrieved using our search criteria, and split based on disorder/search term (see Fig. 1). After initial screening of titles and removing of duplicates, 836 papers were excluded. 2267 papers were excluded due to unrelated articles, not written in English, no abstract available. A further 356 papers were excluded due to unrelated articles, repeated publications, no abstract available, reviews, meta-analysis, meeting abstracts, animal models, studies on rare monogenetic disorders/syndromes, protocols of hiPSC differentiation without including the generation of a disorder- and patient-specific iPSC cell line, study protocols of planned studies without actual results. The final number of papers included in our review was 56, which again was split based on disorder/search term. Six disorder/search terms did not yield any papers after eligibility assessment; personality disorder, antisocial personality disorder, anxiety disorder, substance use disorder, eating disorder and gaming disorder. Synthesised results from each of the other disorders/search terms are described below (see Fig. 1 and Table 1).

**Table 1:** Summary of the iPSC-based neuropsychiatric studies reviewed

	iPSC Generation				
Disorder	Biological replicates (*n*)	Somatic cell origin	Reprogramming Technique	iPSC differentiation	References
ASD	1 patient; 2 controls	Dental pulp cells	Retroviral	NPCs, neuronal cells	Griesi-Oliveira et al. (2015)
	4 patients; 8 controls	Fibroblasts	Retroviral, episomal	Telencephalic neurons	Mariani et al. (2015)
	8 patients; 5 controls	Fibroblasts	Retroviral	NPCs, neuronal cells	Marchetto et al. (2017)
	8 patients; 7 controls	Fibroblasts	Retroviral	Neuronal cells	Amatya et al. (2019)
	3 patients; 3 controls	Fibroblasts	Lentivirus, Sendai virus	NPCs	Moore et al. (2019)
	2 patients; 2 controls	Fibroblasts, CD34 + blood cells	Lentivirus, Sendai virus	NPCs, neuronal cells	Ross et al. (2019)
BD	3 patients; 3 controls	Fibroblasts	Retroviral	Neuronal cells	Chen et al. (2014)
	1 patient; 1 control	Fibroblasts	Retroviral	NPCs, neuronal cells	Bavamian et al. (2015)
	4 patients; 4 controls	Fibroblasts	Sendai virus	NPCs, neuronal cells	Kim et al. (2015)
	2 patients; 2 controls	Fibroblasts	Retroviral	NPCs, neuronal cells	Madison et al. (2015)
	6 patients; 4 controls	Fibroblasts	Sendai virus	Hippocampal dentate gyrus (DG) granule cell-like neurons	Mertens et al. (2015)
	11 patients; 8 controls	Fibroblasts, lymphocytes	Episomal, retroviral, lentiviral	NPCs, cortical interneurons	Tobe et al. (2017)
	6 patients; 3 controls	Adipocytes	Sendai virus	Cortical NSCs	Vizlin-Hodzic et al. (2017)
	2 patients	Fibroblasts	Sendai virus	N/A	Palladino et al. (2018)
	6 patients; 4 controls	Lymphocytes	Episomal	Hippocampal dentate gyrus (DG) granule cell-like neurons	Stern et al. (2018)
	1 patient	PBMCs	Episomal	N/A	Wang et al. (2018a)
	2 patients; 7 controls; 1 isogenic control	Fibroblasts, T-cells, lymphocytes	Episomal, retroviral	Glutamatergic + GABAergic neurons	Ishii et al. (2019)
SCZ	4 patients; 3 control	Fibroblasts	Lentiviral	NPCs	Brennand et al. (2011)
	3 patients; 2 controls	Fibroblasts	Retroviral	Glutamatergic neurons	Pedrosa et al. (2011)
	1 patient; 1 control	Fibroblasts	Retroviral vectors	NPCs	Paulsen et al. (2012)
	3 patients; 2 controls	Hair follicle keratinocytes	Lentiviral	NPCs, glutamatergic neurons	Robicsek et al. (2013)
	2 patients; 2 controls	Fibroblasts	Lentiviral	Neuronal cells	Bundo et al. (2014)
	4 patients; 5 controls	Fibroblasts	Lentiviral	NPCs	Hashimoto-Torii et al. (2014)
	4 patients; 3 isogenic controls	Fibroblasts	Episomal	Forebrain-specific NPCs, cortical neurons	Wen et al. (2014)
	3 patients; 3 controls	Fibroblasts	Episomal and Sendai virus	NPCs	Yoon et al. (2014)
	4 patients; 6 controls	Fibroblasts	Lentiviral	Forebrain NPCs	Topol et al. (2015)
	4 patients; 6 controls	Fibroblasts	Lentiviral	Forebrain NPCs	Brennand et al. (2015)
	1 patient; 1 control	Fibroblasts	Lentiviral	NPCs, glutamatergic neurons	D'Aiuto et al. (2015)
	2 patients; 1 control	Fibroblasts	Sendai virus	NPCs, neuronal cells	Das et al. (2015)
	3 patients; 5 controls	Fibroblasts	Sendai virus	NPCs, neuronal cells, OPCs	Lee et al. (2015)
	1 patient; 4 isogenic controls	Fibroblasts	Lentiviral	NPCs, neuronal cells	Srikanth et al. (2015)
	8 patients; 7 controls	Fibroblasts	Episomal	Neuronal cells	Lin et al. (2016)
	6 patients; 6 controls	Fibroblasts	Episomal	Neuronal cells	Zhao et al. (2015)
	2 patients; 3 controls	Fibroblasts	Episomal	NPCs	Murai et al. (2016)
	4 patients; 10 controls	Fibroblasts	Lentiviral and Sendai virus	NPCs	Topol et al. (2016)
	2 patients; 3 controls	Fibroblasts	Retroviral	Neuronal cells	Toyoshima et al. (2016)
	2 patients	Fibroblasts, lymphocytes	Lentiviral and Sendai virus	Glutamatergic forebrain neurons	Forrest et al. (2017)
	1 patient; 1 control	Fibroblasts	Episomal	Forebrain-specific organoids	Ye et al. (2017)
	2 patients; 2 controls	Hair follicle keratinocytes	Lentiviral	Glutamatergic neurons	Robicsek et al. (2018)
	2 patients; 7 controls; 1 isogenic control	Fibroblasts, T-cells, lymphocytes	Episomal, retroviral	Glutamatergic + GABAergic neurons	Ishii et al. (2019)
ADHD	3 patients	Urothelial cells	Sendai virus	N/A	Sochacki et al. (2016b)
	1 patient	Fibroblasts	Sendai virus	N/A	Jansch et al. (2018)
	1 patient; 3 controls	Hair follicle keratinocytes	Sendai virus	N/A	Re et al. (2018)
	1 patient; 1 control	PBMCs	Sendai virus	N/A	Tong et al. (2019)
Anorexia nervosa	4 patients; 4 controls	Fibroblasts	Retroviral	NPCs, cortical neurons	Negraes et al. (2017)
MDD	6 patients; 3 controls	Fibroblasts	Sendai virus	NPCs, forebrain neurons	Vadodaria et al. (2019a)
	6 patients; 3 controls	Fibroblasts	Sendai virus	NPCs, serotonergic neurons	Vadodaria et al. (2019b)
OCD	1 patient	PBMCs	Episomal	N/A	Wang et al. (2018b)
	1 patient	Urothelial cells	Sendai virus	N/A	Sochacki et al. (2016a)
ASPD	6 patients; 9 controls	Fibroblasts	Sendai virus	Cortical neurons, astrocytes	Tiihonen et al. (2019)
AUD	4 patients; 3 controls	Fibroblasts	Retroviral	Neuronal cells	Lieberman et al. (2012)
	12 patients; 9 controls	Fibroblasts	Retroviral, Sendai virus	Neuronal cells	Lieberman et al. (2015)
	3 controls	Lymphocytes	Sendai virus	NPCs	De Filippis et al. (2016)
	11 patients; 13 controls	Fibroblasts	Retroviral, Sendai virus	Neuronal cells	Lieberman et al. (2018)
Nicotine use disorder	3 patients; 3 controls	Lymphocytes	Sendai virus	Dopaminergic + glutamatergic neurons	Oni et al. (2016)
Opioid use disorder	2 patients; 2 controls	Fibroblasts	Lentiviral	Dopaminergic neurons	Sheng et al. (2016)

*NPCs* Neuronal progenitor cells, *OpCs* oligodendrocyte precursor cells, *ASD* autism spectrum disorders, *BD* bipolar disorder, *SCZ* schizophrenia disorder, *ADHD* attention-deficit/hyperactivity disorder, *MDD* major depressive disorder, *OCD* obsessive compulsive disorder, *ASPD* antisocial personality disorder, *AUD* alcohol use disorder

### Autism-spectrum disorders

Individuals diagnosed with autism spectrum disorder (ASD) show characteristic deficits in social communication and interaction, and often demonstrate restricted, repetitive behaviors and interests (Ivanov et al. 2015). The disorder is highly heritable, with concordance rates in monozygotic twins of 70–90%. A monogenetic cause has been defined for some forms of ASD, however the exact genetic cause is known in only 20–25% of cases (Ivanov et al. 2015). Studies investigating hiPSC models of rare, monogenetic forms and/or syndromic of ASD have previously been reviewed elsewhere (Brito et al. 2018). In non-syndromic cases of autism, there has been little research performed using iPSC-based models. Transcriptome and gene network analyses of iPSC-derived organoids revealed that gene expression involved in cell proliferation, neuronal differentiation, and synaptic assembly was increased in ASD compared to controls (Mariani et al. 2015). Furthermore, the organoids showed abnormally high expression of inhibitory GABAergic interneurons, as well as an accelerated cell cycle. Consistent with these results, increased proliferation of neural progenitor cells has been found in iPSC-derived 2D cultures from ASD patients, along with reduced synaptogenesis leading to abnormal function of neural networks (Marchetto et al. 2017). Corroborating these results, investigation of neuronal activity in iPSC-derived neurons from ASD patients revealed bursting and spike interval parameters that pointed to a significant reduction in dynamical complexity (Amatya et al. 2019). Furthermore, an impairment in iPSC-derived neuronal differentiation has been observed and linked to micro RNA function; specifically the downregulation of miR-1290, which is involved in neural proliferation and differentiation (Moore et al. 2019). More recently, iPSC-derived neural cell models have helped to further characterize newly identified genetic risk genes for ASD, such as *PTCHD-1-AS* (Ross et al. 2019). Alterations in this gene were found to cause synaptic dysfunction, identified using electrophysiological experiments. Additionally, in models using iPSC-derived neural cells with disruptions in the *TRPC6* gene, it was found that altered neuronal differentiation, morphology, and function was associated with dysregulation of CREB phosphorylation (Griesi-Oliveira et al. 2015).

### Bipolar disorder

Bipolar disorder is a primarily episodic disorder which is characterised by alternating manic episodes, and depressed episodes. In between, there are so called euthymic phases with no or only little affective symptoms (Shastry 2005). Bipolar disorder affects 1 to 2% of the world’s population, showing similar incidence rates in both males and females (Hirschfeld et al. 2003). It is a highly familial disorder, with heritability estimates of up to 80% (Craddock and Jones 1999). Recent genome-wide association studies revealed several common risk gene variants to be associated with bipolar disorder (Stahl et al. 2019), and rare genetic variants do not appear to play an important role in the complex genetic architecture of bipolar disorder (Georgieva et al. 2014). The first studies investigating iPSC-derived neuronal cells from bipolar disorder patients were published in 2014. Chen and colleagues reported differential gene expression in iPSC-derived neuronal cells from bipolar patients in comparison to healthy controls (*n* = 3 biological replicates each). Specifically, expression of genes encoding membrane-bound receptors, ion channels and telencephalic neuronal differentiation was significantly increased in the neurons generated from bipolar patients compared to controls. Additionally, in vitro lithium treatment had a significant effect on calcium signaling and electrophysiological properties in bipolar neurons but not in controls (Chen et al. 2014). Bavamian and colleagues compared miR-34a expression in post-mortem brain tissue from medication-naïve bipolar patients and healthy controls with miR-34a levels from directly induced neuronal cells (iNs) and iPSC-derived neuronal cells, from a bipolar son and his unaffected father. In post-mortem cerebellum tissue, iNs and iPSC-derived neuronal cells, miR-34a expression was increased in the bipolar patients in comparison to healthy controls. The effect of miR-34a on previously identified bipolar risk genes was also investigated, and *ANK3*, *DDN*, and *CACNB3* were shown to be targeted and potentially silenced by miR-34a. In a later study, overexpression of m**i**R-34a in vitro was reported to decrease *CACNB3* and *ANK3* gene expression, and alter the neuronal differentiation and morphology of human iPSC-derived neurons from healthy controls (Bavamian et al. 2015). Madison and colleagues derived iPSCs from the fibroblasts of the unaffected father and mother from a bipolar patient and two brothers with bipolar disorder type I. This family was also genotyped for the previously published risk SNPs (single nucleotide polymorphisms) associated with bipolar disorder. All four family members were either homozygous or heterozygous for the bipolar risk genotypes in the *SYNE1*, *ANK3*, *CACNA1C*, *ODZ4* (*TNM4*) and *ZNF804A* genes, of which *ZNF804A* is also a schizophrenia risk allele. The iPSCs were differentiated into CXCR4^+^ NPCs, and abnormalities in the early steps of NPC formation in the bipolar siblings were reported. Additionally, alterations in WNT/GSK3 signaling were observed, suggesting dysregulated ion channel expression in NPCs and neuronal cells (Madison et al. 2015). Mertens and colleagues showed differential responses to in vitro lithium treatment regarding hyperexcitability in iPSC-derived hippocampal dentate gyrus-like neuronal cells from bipolar patients (*n* = 6), generated from clinical lithium responders vs. bipolar lithium non-responders and healthy controls (*n* = 4). Evidence for mitochondrial abnormalities in neurons from bipolar patients was also shown (Mertens et al. 2015, 2016). The finding of hyperexcitability in iPSC-derived hippocampal dentate gyrus granule-cell neurons could be replicated in another study using Epstein-Barr immortalized lymphoblast cells as primary cells from bipolar patients and healthy controls (three lithium-responders, three lithium non-responders and four healthy controls). The iPSC-derived neuronal cells were treated with lithium and the electrophysiological data was used to train a Naïve Bayes (NB) classifier that could predict if a novel bipolar patient was lithium-responsive or non-responsive with an accuracy of up to 98% (Stern et al. 2018). Proteomic differences between cells of lithium responders and non-responders have been investigated in iPSC-derived dorsal anterior forebrain cortical neurons generated from bipolar patients, unaffected family members, a family member affected with major depression and a cell line from a Parkinson’s patient. The results suggested that the molecular lithium-response pathway in bipolar patients may function via CRMP2 (collapsin response mediator protein-2), which acts to modify neuronal cytoskeletal dynamics (specifically dendrite and dendritic spine formation) (Tobe et al. 2017). Kim et al. reported the generation of iPSC lines from 4 bipolar disorder affected and 4 unaffected relatives in an Old Order Amish pedigree that were differentiated into NPCs and further differentiated into cortical neurons in vitro. They found several genes differentially expressed between bipolar patients and healthy controls using microarray analysis, but only in the more mature neuronal cells. The most significantly differentially regulated pathways included RNA metabolic processes, protein trafficking and receptor-mediated signaling (Kim et al. 2015)*.* Vizlin-Hodzic and colleagues used primary adipocytes for generating iPSCs and differentiated them into cortical neuronal cells from six bipolar I patients and three healthy controls. Using RNA-sequencing, the expression of the inflammasome *NLRP2* gene (NLR family, pyrin domain containing 2) was the most significantly differentially regulated gene between BD I and control lines in both iPSC and cortical neurons. Several cytoskeleton- and inflammatory-associated genes, and GABA and dopamine receptor signaling pathways, were also significantly dysregulated in bipolar neuronal cells compared to the control cells (Vizlin-Hodzic et al. 2017).

There have been several studies investigating specific risk gene variants in iPSC-derived neuronal cells from bipolar patients. Ishii et al. compared glutamatergic and GABAergic neurons derived from two bipolar patients carrying a *PCDH15* deletion with seven healthy control cell lines and isogenic controls. In bipolar neurons, decreased MAP2^+^ dendrite length and synapse number were shown (Ishii et al. 2019). Additionally, the generation of a bona fide iPSC cell line of a Chinese bipolar patient carrying the potential risk variants *SLC1A3* rs117588697 A-G/, *MAPT* rs577501443 G-A, *SIRT1* rs12415800 G-A was recently reported (Wang et al. 2018a). In our own lab, we have generated one bone fide iPSC cell lines from a bipolar patient who is a carrier of a risk haplotype in the diacylglycerol kinase eta gene (*DGKH*; rs994856/rs9525580/rs9525584 GAT) and from another bipolar patient who is a carrier of the wildtype haplotype (Palladino et al. 2018). This *DGKH* risk haplotype was previously shown to be associated with bipolar disorder, but also with major depression and adult ADHD, and was observed to have functional effects on brain volume and peripheral gene expression (Kittel-Schneider et al. 2014, 2016; Weber et al. 2011).

### Schizophrenia

Schizophrenia (SCZ) is a highly heritable, severe, and complex psychiatric disorder, that affects ~ 1% of the general population (Owen et al. 2016). Despite high heritability estimates (Hilker et al. 2018; Sullivan et al. 2003) and a century of biological investigation (Ahmad et al. 2018), the genetics underling SCZ remain unclear and no conclusive disease mechanisms have been identified. It is thought that the disorder is highly polygenic (Giegling et al. 2017; Gratten et al. 2014), and GWAS have detected multiple common genetic variants of small effect size that explain up to 50% of variability in genetic susceptibility (Purcell et al. 2014). The first study using an iPSC-based model to investigate SCZ was conducted in 2011 by Brennand et al. who reported decreased connectivity, neurite outgrowth and expression of post-synaptic density protein 95 (PSD95) in SCZ iPSC-derived NPCs (SCZ iPSC-NPCs) (Brennand et al. 2011). Significant alterations were also observed in gene expression relating to glutamate and Wnt signaling. Interestingly, treatment with the antipsychotic loxapine appeared to ameliorate these effects, enhancing neuronal connectivity and rescuing gene expression. However, it should be noted that other structurally related antipsychotic drugs failed to rescue these deficits. Despite this, changes in Wnt signaling in SCZ iPSC-derived NPCs was later replicated in other studies (Topol et al. 2015), as was inhibited NPC growth and migration (Brennand et al. 2015; Lee et al. 2015). Collectively, the studies to date suggest that early neurodevelopmental pathways may be perturbed in SCZ. Mitochondrial (Mt) dysfunction has been consistently implicated in SCZ. Increased extra-Mt oxygen consumption (Paulsen et al. 2012) and reactive oxygen species (ROS) levels in SCZ iPSC-derived NPCs have been observed (Brennand et al. 2015; Paulsen et al. 2012; Robicsek et al. 2013), which could be reversed by treatment with valproic acid. Altered Mt protein expression has additionally been found directly in SCZ iPSCs, which correlated with decreased membrane potential and an uneven cellular distribution of Mt, which is characteristic of impaired function (Robicsek et al. 2013). Perturbed Mt morphology was further observed by other groups, with SCZ iPSC-NPCs containing Mt that were decreased in size, less connected and not as densely placed around the nucleus (Brennand et al. 2015). As proof-of-principle, Robicsek et al. (2018) recently transferred healthy Mt to SCZ iPSCs using isolated active normal mitochondria (IAN-MIT) methodology, which improved membrane potential and Mt distribution (Robicsek et al. 2018). These studies imply that perturbed Mt respiration and morphology combined with increased oxidative stress may play a key role in SCZ, and moreover, that Mt transfer may be a feasible treatment option for reversing these deficits.

Altered micro RNA (miRNA) expression and/or function has been demonstrated in case/control in SCZ-iPSC studies, affecting both individual genes and complex networks. A GWAS-predicted functional SCZ risk SNP in the *MIR137* gene was demonstrated to reduce miR-137 expression during iPSC-derived glutamatergic forebrain neuron differentiation, using isogenic cell lines generated using CRISR-Cas9 technology (Forrest et al. 2017). Decreased miR-9 activity has also been observed in SCZ iPSC-NPCs, concomitant with inhibited radial migration of neurospheres, which could be rescued by subsequent overexpression of miR-9 (Topol et al. 2016). miR-219 is brain-specific and has additionally been reported dysregulated in SCZ, resulting in abnormal neural stem cell proliferation and depletion of the neural stem cell pool (Murai et al. 2016). These effects could be rescued by treatment with a miR-219 inhibitor, suggesting that the miR-219 pathway plays a key role in neural stem cell proliferation and could be a potential therapeutic target. Other miRNAs found to be differentially expressed in SCZ iPSC-based models include miR-34, miR-4449, miR-146b-3p, and miR-23a-5p (Zhao et al. 2015). 22q11.2 deletion is a well-recognized risk factor for the development of SCZ (Marshall et al. 2017), and has been associated with both altered miRNA (Zhao et al. 2015) and gene expression (Lin et al. 2016) in SCZ iPSC-derived neurons. Moreover, 22q11.2 deletion SCZ-iPSCs were observed to have impaired differentiation capacity, smaller neurosphere size and inhibited neurite outgrowth/migration (Pedrosa et al. 2011; Toyoshima et al. 2016). However, a lack of genetically engineered cell models that can rescue these effects means that a candidate causal gene in this deleted chromosome region has yet to be identified. In contrast, a putative causal gene has been identified in 15q11.2 deletion, another risk factor for SCZ (Stefansson et al. 2008). SCZ iPSC-NPCs were found haploinsufficient for the cytoplasmic FMR1 interacting protein 1 (*CYFIP1*) gene, which is located in this deleted region (Yoon et al. 2014). The cells also demonstrated deficiency in apical polarity and adherens junctions, implying dysregulated neuronal maturation. Subsequent studies supported these findings, with decreased *CYFIP1* and altered morphology additionally observed in 15q11.2 deletion iPSC-derived neurons (Das et al. 2015). The rare *DISC1* mutation is another well-replicated risk gene for SCZ (Millar et al. 2000) that has been investigated using iPSC-based cell models. iPSC-derived neurons containing mutated *DICS1* were found to have impaired synaptic vesicle release, which could be induced in control cells and reversed in patient cell lines using TALENs to specifically edit the *DISC1* gene, strongly implicating a role of this gene in synaptic dysfunction (Wen et al. 2014). TALEN and CRISPR-Cas9 editing was further used to mutate *DISC1* directly in iPSCs, resulting in abnormal neural differentiation and dysregulated Wnt signaling, consistent with previous findings in SCZ (Srikanth et al. 2015). Moreover, in the previously described study by Murai et al. (2016) which showed increased miR-219 expression and altered proliferation, *DISC1* mutations were also found to be present in SCZ iPSC-derived neural stem cells (Murai et al. 2016). Other studies have since replicated this result (Ye et al. 2017), suggesting a role for *DISC1* in the regulation of proliferation and providing a potential pathomechanism for SCZ development. Reelin (*RELN*) is another gene involved in brain developmental processes that has been associated with SCZ, with exonic deletions specifically reported (Costain et al. 2013; Ishii et al. 2016). A recent study also employed the use of genomic editing, comparing glutamatergic and GABAergic iPSC-derived neurons from a SCZ patient with *RELN* deletion to those from isogenic controls genetically engineered to possess a *RELN* deletion (Ishii et al. 2019). The resulting phenotypes were similar, with dendrite-shortening and decreased synapse numbers observed, further implicating this gene in SCZ development.

There are several environmental risk factors for SCZ, such as prenatal starvation (St Clair et al. 2005; Susser et al. 1996; Susser and Lin 1992) and infection-induced maternal immune activation (MIA) (Brown and Derkits 2010; Estes and McAllister 2016), that are starting to be investigated using iPSC-based models. SCZ iPSC-NPCs were observed to differ in their sensitivity to subthreshold environmental stressors such as alcohol and mercury, as inferred by differential heat shock factor 1 (HSF1) activation (Hashimoto-Torii et al. 2014). However, the mechanisms underlying this different susceptibility were not further investigated. Increased copy number of LINE-1 retrotransposons has been observed as a consequence of MIA in animal models, and was also observed in SCZ iPSC-derived neurons from 22q.11 deletions patients, suggesting prior infection (Bundo et al. 2014). SCZ iPSC-derived neurons have been directly infected with the herpes simplex virus type 1 (HSV-1), and alterations in expression of genes involved in glutamatergic signaling and Mt function were consequently observed (D'Aiuto et al. 2015). Despite these initial studies, comprehensive iPSC-based studies investigating the effect of MIA on SCZ-relevant molecular mechanisms are still lacking (Balan et al. 2019). Moreover, the iPSC-based models used so far are simplistic and not reflective of the in vivo scenario, where viral response requires the concerted effort of neuronal, immune and glial cells (D'Aiuto et al. 2015). To overcome this, future studies should aim to use co-culture systems, which utilize a number of cell types and permit cell–cell interaction (Balan et al. 2019).

There are several limitations to using iPSC-based models for SCZ. Even after prolonged cell culture, iPSC-derived neurons lack the myelination required for maturation and still resemble fetal stage neurons, therefore caution is needed when extrapolating results from iPSC-based models to adolescent/adult SCZ patients (Ahmad et al. 2018; Balan et al. 2019). Additionally, SCZ generally has its onset in adolescence (Häfner et al. 1994), further questioning the validity of using immature neurons to investigate its etiology. A further problem is that of sample heterogeneity, with iPSCs generated from different subtypes of SCZ shown to demonstrate differential results, for example in undifferentiated (Brennand et al. 2011) versus paranoid SCZ (Robicsek et al. 2013). This has also been observed in SCZ iPSCs derived from treatment-responsive versus treatment-resistant patients (Grunwald et al. 2019; Nakazawa et al. 2017; Paulsen et al. 2012). Moreover, patient genetic background has been found to have opposing effects on miRNA expression (Ahmad et al. 2018). In the future, sample heterogeneity may be counteracted by selecting ‘general’ SCZ samples based on phenotype and/or polygenic risk score, and utilizing familial samples to reduce the confounding effect of differential genetic background (Balan et al. 2019).

### Attention-deficit/hyperactivity disorder

Attention-deficit/hyperactivity disorder (ADHD) is a neurodevelopmental disorder characterised by inappropriate and persistently high levels of inattention, locomotor hyperactivity, and impulsivity (American Psychiatric Association 2013). ADHD is one of the most common neurodevelopmental disorders of childhood, and shows a high persistence of symptoms into adulthood (approximately two-thirds) (Faraone et al. 2006). Impairment by ADHD symptoms entails a considerable socioeconomic burden for affected individuals (Gjervan et al. 2012). It is well established that heritability rates for ADHD rank amongst the highest of all mental disorders at ~ 75% (Faraone and Larsson 2019), similar to schizophrenia (Sullivan et al. 2003), bipolar disorder (McGuffin et al. 2003), and autism spectrum disorders (Sandin et al. 2017). As with most common psychiatric disorders, ADHD demonstrates a polygenic inheritance pattern with a multiplicity of genes involved, most of them contributing only a small proportion to the overall genetic variance of the disorder (Faraone and Larsson 2019). Early genetic studies focused on the investigation of monoaminergic candidate genes (Cook et al. 1995; Kustanovich et al. 2004; Swanson et al. 1998). Several disease-associated candidate genes have also been identified, such as *ADGRL3* (= *LPHN3*) (Arcos-Burgos et al. 2010), *GRM5* (Elia et al. 2012), and *NOS1* (Weber et al. 2015), which play fundamental roles in excitatory glutamatergic neurotransmission (Lesch et al. 2012). Furthermore, 12 significant loci revealed in a recent ADHD GWAS were found to contain several promising genes, such as *ST3GAL3*, *SORCS3*, and *FOXP2*, all of which show strong expression in the central nervous system (CNS) (Demontis 2019). These genes are additionally involved in neurodevelopmental processes, further supporting a possible role in ADHD etiology (Hu et al. 2011) (Breiderhoff et al. 2013) (Yoo et al. 2015) (Chen et al. 2016). Several groups have reported the generation of iPSCs derived from ADHD patients (Jansch et al. 2018; Re et al. 2018; Sochacki et al. 2016b; Tong et al. 2019). However, functional analyses of these new cell lines were not conducted, and therefore form the next logical step of investigation. These studies should integrate hypothesis-driven and hypothesis-free gene expression experiments, under both basal and drug-treatment conditions, to assess possible modes of drug action. A number of studies have already addressed the effects of psychostimulants in cell culture, however these studies relied on animal cell culture models, non-neuronal human cell models and/or transfection systems for overexpression of the dopamine (DAT) or norepinephrine transporter (Grünblatt et al. 2013; Schwarz et al. 2014). For example, in a murine stem cell model, it was shown that methylphenidate treatment in vitro could enhance neuronal differentiation (Bartl et al. 2013). Furthermore, differences in axonal outgrowth and synaptic connectivity between patient and healthy control iPSC-derived neurons could be assessed with microfluidic chips in future studies. In summary, ADHD iPSC-based models offer the unique potential to close the gap between basic genetic and neural network associations, by identifying potential disease-modifying cellular pathways and adding valuable information to the endophenotypic finger print of ADHD (Castellanos and Tannock 2002). However, research has been inhibited by the lack of functional characterisation of these models, which should now be the priority for researchers working in this field.

### Eating disorders

To date, there has only been one study conducted using iPSCs to investigate the etiology of an eating disorder (ED). The authors focused on anorexia nervosa (AN), which is a multifactorial neurodevelopmental disorder that affects ~ 1% of the population (Smink et al. 2012). The disorder presents with distorted body image and severe caloric restriction, usually as a result of a highly anxiogenic response to food, which ultimately leads to emaciation and/or death (Sharan and Sundar 2015). AN has the highest mortality rate of all psychiatric diseases (Arcelus et al. 2011), and there are currently no highly efficient treatments (Bulik et al. 2007; Watson and Bulik 2013). This is largely due to a lack of understanding of disease etiology, and research lags behind other psychiatric diseases. This is likely due to the traditional view that EDs are non-biologically based problems that are caused by vanity and poor parenting, and only occur in specific groups of people (O'Hara and Smith 2007). However, studies have suggested that 50–75% AN risk is due to genetics (Bulik et al. 2005), although no genome-wide association study has so far been powered enough to identify specific genes significantly associated with the disease (Brandys et al. 2015; Wang et al. 2011). Negraes et al. (2017) generated iPSCs from 4 AN patients and 4 healthy controls, expanding two clones from each sample (Negraes et al. 2017). To reduce sample heterogeneity, all AN patients included had similar severity of symptoms and medical/behavioral consequences from AN symptoms. However, some patients also used compensatory behavior such as purging. AN binge-eating/purging has been recognized as a clinically relevant subtype, demonstrating higher levels of core ED psychopathology and more severe cognitive impairments, and therefore may cause significant sample heterogeneity (Reas and Rø 2018; Tamiya et al. 2018). The authors were able to successfully generate iPSC-derived cortical neurons, with no differences in differentiation capability observed. This was supported by RNA sequencing data (RNASeq), which showed no changes in genes related to neural development/differentiation, suggesting that developmental anomalies may not be present in AN patient brains. RNASeq also revealed that in contrast to previous genetic studies (Gervasini et al. 2013; Kaye et al. 1988, 2005; Levine et al. 2000; Phillipou et al. 2014; Rask-Andersen et al. 2010), there were no changes in serotonin or dopamine-related genes in AN patient neurons. However, 361 differentially expressed genes were observed. 13 candidate genes that showed highly different expression were selected and confirmed by RT-qPCR (Negraes et al. 2017). Tachykinin receptor 1 (*TACR1*) was the mostly significantly upregulated gene in AN patient neurons, and increased protein expression was also observed. This gene has already been associated with psychiatric disorders such as anxiety (Muñoz and Coveñas 2014) and addiction (Schank 2014). However, in animal models this gene increases the risk of high BMI, which is at odds with AN presentation (Pillidge et al. 2016). Therefore it remains unclear as to what, if any role, tachykinin signaling may play in AN etiology. The diacylglycerol kinase gamma (*DGKG*) gene was additionally found upregulated in AN neurons, which has previously been associated with obesity (Cheung et al. 2010) and chronic stress (Lisowski et al. 2013), suggesting it may potentially play a role in the development of AN. Several other genes also found differentially expressed were connective tissue growth factor (*CTCF*) and tudor domain containing 10 (*TDRD10*), which are involved in normal ovarian follicle development/ovulation (Nagashima et al. 2011) and gametogenesis (Hosokawa et al. 2007) respectively. However, as amenorrhea and infertility commonly result from AN (Bulik et al. 2005; Katz and Vollenhoven 2000), these changes in gene expression are more likely to be consequential than causative.

### Major depressive disorder

Major depressive disorder (MDD) is the most common psychiatric disorder with a life time prevalence of 10% worldwide (Wittchen et al. 2001). Core symptoms are depressed mood, decreased energy and lack of interest. Suicide is a major complication of MDD, and women suffer about twice as often than men (Wittchen et al. 2011). MDD is a highly heterogeneous disorder, and genetic factors are thought to contribute ~ 50% to disease etiology (Ormel et al. 2019). Recent genome-wide association studies reported only a few replicated common risk gene variants associated with major depression (Ormel et al. 2019). With regards to unipolar or MDD, the focus of iPSC-based studies has been on cells generated from patients with therapy-resistant depression, with our literature search revealing two currently published studies by the same research group. Vadodaria and colleagues generated iPSC-derived forebrain neurons from three excellent selective-serotonin-reuptake-inhibitor (SSRI) responder patients, three non-responder patients and three healthy controls without a history of depression. Calcium signaling was measured as a marker for neuronal activity using the calcium indicator dye Fluo-4. There was no significant difference between the three groups at baseline, however after 5-Hydroxytryptamine (= serotonin, 5-HT) in vitro treatment non-responder forebrain neurons displayed significantly higher activity compared to responder and healthy controls. After further analysis, it was found that SSRI-non-responder neurons displayed 5-HT-induced hyperactivity via upregulated 5-HT2A and 5-HT7 receptors (Vadodaria et al. 2019a). In contrast, serotonergic neurons from three SSRI-responders and three non-responders did not demonstrate significant differences in 5-HT release/reuptake, or in genes related to 5-HT signaling. However, non-responder serotonergic neurons did appear to have altered neurite growth and morphology compared to healthy controls and SSRI-responders. Transcriptome analysis of these neurons showed dysregulated protocadherin alpha gene expression (*PCDHA6* and *PCDHA8*) in non-responders compared to responders and healthy controls. Additionally, knockdown of *PCDHA6* and *PCDHA8* in HEK cells and serotonergic neurons was found to alter neurite length, suggesting that differences in non-responder serotonergic neuron length may be mediated by decreased *PCDHA6* and *PCDHA8* gene expression levels (Vadodaria et al. 2019b).

### Obsessive–compulsive disorder

The concept of obsessive–compulsive disorder (OCD) has changed during recent years, most notably with the switch of diagnostic categorization from an anxiety disorder in DSM-IV (American Psychiatric Association 2000) to its own diagnostic section of “obsessive–compulsive and related disorders” in DSM-5, which also comprises skin picking disorder, hair pulling disorder, hoarding, and body dysmorphic disorder (American Psychiatric Association 2013). Lifetime prevalence in the United States is estimated to be 2.3% (Ruscio et al. 2010). (Epi)genetic data, functional imaging studies, and neuropsychological data point to a complex interplay between environmental and biological factors in OCD etiology (Pauls et al. 2014), with an estimated heritability of ~ 40% in adult- and up to 65% in childhood-onset OCD (Abramowitz et al. 2009; Taylor 2011). Childhood-onset OCD exhibits a higher genetic load with increased familial risk in comparison to adult-onset OCD (Nestadt et al. 2000; Pauls et al. 1995). A neural network between cortical regions and the basal ganglia, called the cortico-striato-thalamo-cortical (CSTC) circuit, has also been repeatedly implicated in OCD (Graybiel and Rauch 2000; Posner et al. 2014). A meta-analysis of the two previous OCD GWAS showed no genome-wide significant findings, but amongst the top 3 haploblocks was a region comprising the *GRID2* gene (IOCDF-GC and OCGAS 2018). Further evidence for an involvement of glutamatergic neurotransmission in OCD comes from magnetic resonance spectroscopy studies, which suggested increased glutamatergic activity in the striatum (Naaijen et al. 2015). The pharmacological effectiveness of SSRIs implies that serotonergic neurotransmission also plays a role in OCD etiology, and a meta-analytic review of the literature revealed disease-associated variants in both the serotonin transporter (5-HTTLPR) and the serotonin receptor gene *HTR2A* (Taylor 2013). To date, no functional studies using iPSC-derived cell lines from patients with OCD have been published. One group reported the generation of an iPSC line from a 32-year-old male OCD patient from blood cells, and showed that three SNP variants in OCD candidate genes remained unchanged during reprogramming (Wang et al. 2018b). Another group generated an iPSC line from urothelial cells derived from a 29-year old male patient with early-onset OCD and comorbid skin picking and body dysmorphic disorder (Sochacki et al. 2016a). Urothelial cells are a promising source for the generation of iPSCs, as these cells exhibit comparably high reprogramming efficiencies (Drozd et al. 2015; Zhou et al. 2011) and as a non-invasive method might be particularly suitable for psychiatric patients with contamination or needle fears. The development of cerebral organoids needs further progress before complex circuitry of interest, such as the CSTC loop, can be effectively mimicked in vitro. Until then, the focus should be on straightforward strategies for the investigation of alterations in potentially relevant neurotransmission systems to gain new insights into OCD-related cellular alterations. To this end, protocols for the differentiation of both serotonergic (Lu et al. 2016) and glutamatergic neurons (Cao et al. 2017; Gunhanlar et al. 2018) from iPSCs can be used. Additionally, as the overall heritability of OCD is only moderate selection of study subjects will be critical to obtain meaningful insights into cellular mechanisms.

### Personality disorders

Personality disorders (PDs) are classified as ‘a pervasive pattern of thought, feeling and behavior that characterize an individual’s unique lifestyle and mode of adaptation, which deviates markedly from the expectations of the individual’s culture’ (American Psychiatric Association 2000). Examples include borderline, paranoid, narcissistic and obsessive–compulsive PDs (Angstman and Rasmussen 2011). Onset is usually during adolescence or early adulthood, symptoms remain stable over time, and lead to severe impairment and distress (Angstman and Rasmussen 2011). Although the etiology of PDs is still relatively unknown, studies have revealed a moderate to strong genetic contribution, with heritability estimated between 30 and 80% (Fontaine and Viding 2008). Genetic studies have so far been sparse, although candidate genes coding for serotonergic and dopaminergic neurotransmitters have been proposed (Ma et al. 2016; Reichborn-Kjennerud 2010). There is currently only one published study using iPSC-based models to investigate PD pathomechanisms. Tiihonen et al. (2019) focused on antisocial personality disorder (ASPD), of which no underlying molecular pathways are known. It is characterised by aggression, hostility, callousness, manipulativeness, deceitfulness, impulsivity, and its most severe symptom; psychopathy (Tiihonen et al. 2019). ASPD has a prevalence rate of 1–3% in the general population, which increases to 40–70% in prison populations. This study generated iPSC lines and cortical neurons from six ASPD violent offenders, three nonviolent substance abusers (to control for the potential confounding variable of substance abuse), and six control subjects. Several genes were identified to have significantly altered expression in ASPD violent offenders, including the ribosomal (*RPL10P9*) pseudogene, zinc finger protein 132 (*ZNF132*), cadherin 5 (*CDH5*) and opioid receptor delta 1 (*OPRD1*) genes. Moreover, expression of these genes significantly correlated with psychopathy score. Interestingly, all 4 genes have previously been associated with autism (Chiocchetti et al. 2011; Klauck et al. 2006; O'Roak et al. 2012; Redies et al. 2012; Wang et al. 2017), which the authors propose might contribute to the emotional callousness and lack of empathy observed in psychopathic violent offenders. Proteomic analysis showed that the largest effect sizes were observed for opioid-binding protein/cell-adhesion molecule (OPCML), with the highest expression found in ASPD violent offenders, supporting the *OPRD1* transcription data. These results support previous research which suggested that a deficient endogenous opioid system contributes to ASPD (Bandelow and Wedekind 2015).

### Substance use disorders

Substance use disorders (SUDs) rank among the most common psychiatric syndromes and are often characterised by psychosocial dysfunction, unemployment, social decline and somatic or psychiatric comorbidities resulting in a significantly increased mortality (Rehm and Shield 2019). A variety of studies have been performed on the genetic component to SUDs, mainly alcohol use disorder (AUD), and found the heritability of AUD to be ~ 49% (Verhulst et al. 2015). In linkage and genome-wide association studies (GWAS), genes involved in alcohol metabolism such as alcohol dehydrogenase (*ADH*) and aldehyde dehydrogenase (*ALDH*) have been identified as major risk variants in the development of AUD (Frank et al. 2012; Takeuchi et al. 2011; Tawa et al. 2016). The limited number of studies using iPSCs to analyze the biological effects of alcohol on neural cells includes an investigation by Lieberman et al., who found that chronic alcohol exposure lead to a compensatory upregulation of NMDA receptors in iPSC-derived neural cells from genetically heterogeneous AUD patients, but not in control cell lines (Lieberman et al. 2012). This effect suggests that heritable traits might contribute to the development of tolerance to the inhibitory effects of alcohol on NMDA receptor activity in AUD patients (Lieberman et al. 2012). In a subsequent study, the AUD-associated risk allele rs279858*C of *GABRA2*, which encodes for the alpha subunit of the GABA_A_ receptor, was identified to lead to significantly lower receptor expression levels in iPSC-derived neural cell cultures (Lieberman et al. 2015). As the authors could not replicate this result for protein levels in postmortem cortices, they hypothesized that the increased risk for AUD in individuals carrying the polymorphism might result from developmental processes (Lieberman et al. 2015). Additionally, after 21 days alcohol exposure, gene expression of *GABRD* (another GABA_A_ subunit) was upregulated in iPSC-derived neural cells from genetically heterogeneous AUD patients compared to healthy controls (Lieberman et al. 2018). In a different investigation, De Filippis et al. hypothesized that neuronal inflammation might be involved in the pathophysiology of AUD. They demonstrated how alcohol exposure altered mitochondrial and lysosomal functions, and increased sensitivity to oxidative stress, which ultimately lead to activation of the inflammasome pathway (De Filippis et al. 2016). However, this was solely shown in iPSCs derived from healthy individuals, which limits conclusions on the impact of neuronal inflammation in genetically predisposed AUD patients.

Besides AUD, there have been first promising reports on establishing iPSC research for the investigation of nicotine and opioid use disorders. Oni et al. discovered that the risk allele rs16969968 of *CHRNA5* (encoding for the nicotinic receptor alpha 5), which is associated with nicotine addiction, lead to increased response to nicotine and rapid receptor desensitization (Oni et al. 2016). Similar findings have been obtained in iPSC-derived dopaminergic neurons from patients suffering from opioid use disorder, where a variable number tandem repeat (VNTR) polymorphism in the human dopamine transporter *hDAT* resulted in increased protein expression (Sheng et al. 2016).

These few studies must be considered preliminary groundwork for the various possibilities that the use of iPSCs offers in trying to elucidate the neurobiological background of SUDs. Their results however have to be evaluated carefully, as some included iPSCs obtained from mixed populations with an unknown, genetically heterogeneous background. Others recruited only a very limited number of individuals as cell donors, such as* n *= 3 (De Filippis et al. 2016; Oni et al. 2016).

## Discussion and conclusions

There can be no doubt that the advent of iPSC technology has facilitated significant progress in our understanding of neuropsychiatric disease. However, there remains a number of limitations which must be considered, as well as points of discussion on the best procedures for iPSC use.

The process of reprogramming has been shown to erase the epigenetic memory of cells, which presents a problem for both psychiatric disorders known to have an age of onset later than childhood (e.g. SCZ) (Häfner et al. 1994) and those which are thought to be significantly influenced by environmental risk factors, which facilitate their effects via epigenetic marks (Soliman et al. 2017). Transgenerational epigenetic inheritance due to external factors such as malnutrition and stress is gaining traction as an important risk factor in the development of psychiatric disease, and therefore constitutes an important area of study (Yehuda et al. 2016). One possible solution to overcome the erasure of epigenetic marks is via transdifferentiation, which is the process of directly inducing neuronal cells from fibroblasts, without the intermediate generation of iPSCs (Pfisterer et al. 2011). Bypassing reprogramming in this manner has been shown to reduce disruption to the epigenomic landscape, thereby allowing the production of neuronal cells at a ‘pathogenic age’ (Mertens et al. 2018). Alternatively, if a disease-associated epigenetic mark is already known, there is now the possibility to modify that mark directly in iPSCs using CRISPR-Cas9 technology (Xie et al. 2018).

iPSCs and many iPSC-derived cells are grown as a homogenous 2D monolayer, which although provides numerous advantages (such as ease of downstream analysis, simple protocols, highly producible) (Duval et al. 2017; Ho et al. 2018), also has several disadvantages. Neuronal cell-only cultures are inhibited by the absence of other cell types, limiting their ability to recapitulate in vivo aspects such as network functionality, neuronal development and synaptic pruning (Silva and Haggarty 2019). Other physiological brain characteristics, including spatial organisation and extracellular matrix interactions, are also absent. iPSC-derived neuronal cultures therefore cannot be used to study overall brain function, particularly not at the level of neural circuits (Nakazawa et al. 2019). Culturing in a 2D monolayer additionally results in an altered, flat cell morphology, and cell–cell interactions are limited to adjacent cells only (Logan et al. 2019). Recent studies have also reported that the overall quality of the 2D culture (e.g. cell health and differentiation capacity) can be easily effected by various factors such as individual handling of cells, disease status of the donor, differentiation protocols and cell seeding density (Duval et al. 2017). This has resulted in many researchers questioning the validity of studying a single 2D monolayer cell type in isolation, and some believe that the molecular mechanisms underlying drugs responses and physiological pathways simply cannot be accurately modeled in this manner (Ishii et al. 2019). Taken together, the limitations of 2D culture result in low predictive power, warranting caution when extrapolating in vitro results to an in vivo scenario. Co-culture, 3D- and organoid models represent an opportunity to address some of the limitations associated with 2D culture; however, these systems are also not without their disadvantages. For an in-depth review of these newer models see Logan et al. (2019) (Logan et al. 2019). An additional issue is the fact that as well differentiated 2D neuronal cells as brain organoids cannot be differentiated into very mature stages which further limit their usefulness as a model for an adult brain. However, regarding neurodevelopmental disorders like ASD, ADHD and also the prodromal stages of bipolar disorder and schizophrenia that start in childhood as well, we are still convinced that insights to molecular pathomechanisms could be drawn from those immature models.

One of the endemic problems for the use of iPSC models is that the generation of multiple cell lines can be time-consuming, costly and labor-intensive (Quadrato et al. 2016; Soliman et al. 2017). This has resulted in low biological replicate numbers being analyzed, with some studies publishing findings on as little as one biological replicate (Ishii et al. 2019). This presents a problem as it questions the replicability and therefore the validity of findings. Increased biological replicates are needed to minimize sample variability, which has been suggested to vary greatly in iPSC lines (Studer et al. 2015). However, exactly how large the cohort size should be remains uncertain. A recent study by Schwartzentruber et al. (2018) shed some light on this matter, using a large cohort size of 123 biological replicates to analyze molecular and functional variation between iPSC-derived sensory neurons (Schwartzentruber et al. 2018). Their results suggested that between 20 and 80 individual iPSC lines are required to detect the effects of regulatory variants with a moderate-to-large effect size. Although useful knowledge, this now presents researchers with the issue of how to generate such large cohort sizes in a timely, reproducible, yet cost-effective manner. Additional suggestions for decreasing the number of required biological replicates have been proposed by Hoekstra et al. They suggest that patient donors should be selected based on either being carriers of more rare risk gene variants, or possessing a high load of a specific polygenic risk score. They also recommend the use of isogenic controls using CRISPR/cas9 technology, studying affected and non-affected family members, and investigating phenotypically more homogenous patients and controls (Hoekstra et al. 2017).

Replicability of results is further complicated by the variability seen between clones originating from the same iPSC line. When generating iPSCs most protocols recommend that individual colonies demonstrating ESC-like morphology are manually picked and cultured as clones, as they represent successfully reprogrammed cells and therefore increase iPSC homogeneity (Meissner et al. 2007; Pfannkuche et al. 2010). It is therefore current practice to pick and culture multiple clones from one iPSC donor, and many published studies using iPSC-based models present results originating from two or more clones. Although well-intended, the analysis of more than one clone per iPSC line may actually be creating more problems than it solves, due to the variability arising from technical sources such as researcher-subjective colony selection (Chan et al. 2009). A recent study by Germain and Testa (2017) investigated the potential effects of clone variability on iPSC-based disease modeling (Germain and Testa 2017). Using large transcriptomic datasets, they revealed that the use of more than clone per donor increased the detection of spurious differentially expressed genes and the false discovery rate, thereby decreasing the robustness of data. Moreover, this result did not change even when a higher number of biological replicates was used. These results therefore question the current widespread habit of using multiple clones per iPSC donor, a practice that is additionally entrenched into the guidelines of several journals (e.g. Stem Cell Reports). Possible solutions could be (1) to continue to use multiple clones per line, but account for their interdependence in the statistical analysis, (2) use isogenic controls from the same donor, or (3) use only one clone per donor but increase the overall number of biological replicates (Germain and Testa 2017).

A further option could be to use cultures derived from ‘mixed’ clones, which are derived from several initial colonies combined. Bona fide iPSCs have escaped replicative senescence, demonstrating high proliferation rates, and therefore are likely to outgrow non- or only partially reprogrammed cells during culture expansion (Koch et al. 2013; Lapasset et al. 2011). One study investigating this possibility reported that after several passages no significant differences could be observed in morphology, pluripotency, or gene expression profiles between mixed cultures and clonally-derived cultures (Willmann et al. 2013). The concept of using mixed cultures is attractive, as it would help facilitate the standardization and automaton of iPSC generation, allowing for high throughput operation. Further studies are needed to determine whether manual clonal selection is truly necessary for homogenous iPSCs, or whether mixed cultures will suffice. Moreover, there are now technological options for obtaining highly homogenous iPSC cultures without the need for clonal selection, such as the use of magnetic beads for cell selection/sorting (Gao et al. 2018; Yang et al. 2015). It is therefore possible that the act of manually picking individual colonies and publishing results from multiple iPSC clones may become redundant.

One last solution for decreasing data variability, increasing cohort size and consequently increasing data validity, is the use of newly developed iPSC banks. Such initiatives are aimed at generating and collecting a large number of iPSC lines, purely for the purpose of scientific research. Most are non-profit and government-funded, due to the scale and influence required (Huang et al. 2019). These banks allow researchers to increase biological replicate numbers in a time- and cost-effective manner. Moreover, iPSC lines submitted to the banks are stringently checked for quality and iPSC status, in line with guidelines set out by the Global Alliance for iPSC Therapies (Sullivan et al. 2018). For example, the European Bank for induced pluripotent Stem Cells (EBiSC) has established standard operation procedures (SOPs) to provide quality assurance and quality control, tightly regulating processes such as iPSC generation and maintenance. Consequently, data obtained from using iPSC bank cultures may be highly comparable, with decreased variability. For more information, a comprehensive review of current iPSC banks has recently been published by Huang et al. (2019).

An additional advantage of using cell lines from iPSC banks is that the samples provided are usually connected to a wealth of clinical information regarding the donor. Such data can include psychometric assessments, fluid biomarker sampling, and structural, functional and molecular neuroimaging (Chhatwal et al. 2016; Ghetti et al. 2015; Jack and Holtzman 2013; Jack et al. 2013; Marquie et al. 2015). Access to this type of information will allow for the implementation of integrative approaches, providing a rich dataset from which to interpret in vitro iPSC-based results. Studies utilizing clinically informed iPSC models have already started to reveal that dysfunctional mechanisms observed in the patient in vivo can also be modeled in vitro, demonstrating the validity of using this approach. Moreover, these results suggested that disruption to molecular pathways may occur much earlier in disease development than previously thought (Silva and Haggarty 2019). However, despite efforts to promote data sharing in the academic, industry and funding sections, there is still a reluctance to share important results. This is particularly seen in clinical trials, where up to a third of trials remain unpublished up to 5 years after completion (Stefaniak et al. 2017). Such lack of dissemination inhibits the information available to the scientific community, wasting precious resources and hindering progress, and therefore open science in the field of iPSC research should be encouraged in the future.

As mentioned in the introduction, psychiatric diseases are usually highly polygenic disorders, where multiple SNPs convey a small effect size and act in concert to contribute to disease risk (International Schizophrenia et al. 2009). Identifying significantly disease-associated SNPs therefore requires large sample numbers, which as discussed, can be difficult to obtain for iPSC models. To address this problem, a slightly altered approach to study design could be considered. Instead of the classical ‘healthy control versus disease’ design, researchers could instead aim to use the concept of endophenotypes or polygenic risk scores (PRS); or even a combination of the two. Endophenotypes are measurable traits that exist somewhere along the spectrum between disease and distal genotype, falling under categories such as cognitive, biochemical and neuroanatomical, and are proposed to consist of a simpler genetic component than the complex psychiatric disease itself (Gottesman and Gould 2003). PRS measures the risk of an individual developing a certain disease, based on their genetic makeup (Wray et al. 2007). Using GWAS data, each individual disease-associated SNP is assigned a weight based on their effect size, and the total number of SNPs combined with their weights is summed to calculate an individual’s PRS. This approach has already revealed that PRS can significantly predict endophenotypes in disease and control settings, such as cognition (Richards et al. 2019) and impulsivity (Hari Dass et al. 2019). Moreover, PRS can also identify specific pathways which are involved both individually and cross-disorder. For example, certain SNPs existing in calcium signaling genes have now been associated with several different psychiatric diseases, suggesting these genes may have a pleiotropic effect on psychopathology (Cross-Disorder Group of the Psychiatric Genomics Consortium 2013). PRS can therefore be modeled in vitro using iPSC-based models derived from donors for whom sequencing data is available, and consequent functional outcomes analyzed (e.g. synaptic signaling). Moreover, gene editing can be used to manipulate several SNPs simultaneously (Cong et al. 2013; Minkenberg et al. 2017), allowing the creation of isogenic controls based on PRS. Using these approaches will augment the ability of researchers to capture significant molecular changes pertaining to pathomechanisms, providing valuable into disease etiology and potentially revealing novel drug targets.

To summarize, the use of iPSC-based models has greatly contributed to our understanding of the complex etiology of psychiatric diseases, providing a model that is more relevant to the in vivo setting and increasing validity of results. Of course, no method is without its limitations, and the sole use of iPSC cultures to understand mental illness has several. However, these limitations can be seen as areas to improve upon, rather than true limitations (Logan et al. 2019). In this systematic review, we have not only provided the reader with an insight into the investigation of less well-studied psychiatric disorders using iPSCs, but have also provided several possible avenues to explore to increase the potential of current iPSC methods. We hope that implementation of such ideas will aid the discovery of robust novel findings, facilitating valuable insight into disease etiology and informing drug development. The future of iPSC technology appears bright, and by association, so does the future of psychiatric research.
